# Infection with *Clostridioides difficile* ribotype 046 in a paediatric liver transplant patient

**DOI:** 10.1099/acmi.0.000268

**Published:** 2021-10-20

**Authors:** Hildenia B. R. Nogueira, Cecília L. Costa, Conceição S. Martins, Maria Luana G. S. Morais, Carlos Quesada-Gómez, Cibele B. M. Carvalho, Eliane de Oliveira Ferreira, Gerly Anne de Castro Brito

**Affiliations:** ^1^​ Albert Sabin Children’s Hospital, Fortaleza, Ceara, Brazil; ^2^​ Department of Morphology, Faculty of Medicine, Federal University of Ceara, Fortaleza, CE, Brazil; ^3^​ University of Fortaleza, CE, Brazil; ^4^​ Laboratory of Bacteriology, Department of Pathology, Faculty of Medicine, Federal University of Ceara, Fortaleza, CE, Brazil; ^5^​ Facultad de Microbiología and Centro de Investigación en Enfermedades Tropicales, Universidad de Costa Rica, San José, Costa Rica; ^6^​ Federal University of Rio de Janeiro, Institute of Microbiology Paulo de Góes, Laboratory of Anaerobic Biology, Rio de Janeiro, Brazil

**Keywords:** *Clostridioides difficile *infection, liver transplant, molecular characterisation, paediatric

## Abstract

*
Clostridioides difficile* causes nosocomial diarrhoea associated with antibiotic use and immunodeficiency. Although the number of paediatric *C. difficile* infections (CDIs) has increased worldwide, there are few studies on the molecular characterization of strains causing CDIs among children. We report the clinical features and strain molecular characterization of a CDI in a female child with a history of liver transplantation at 7 months of age. This is the first report of the 046 ribotype causing paediatric diarrhoea.

## Introduction

*Clostridioides difficile* is an anaerobic Gram-positive spore-forming bacillus that is usually spread via the faecal–oral route, and is a primary cause of antibitioc-associated diarrhoea in hospitalized patients [1, 2].

*C. difficile* has increasingly been reported as an important pathogen in children in recent years [3–5]. This pathogen is responsible for a broad spectrum of diseases in children, ranging from secretory diarrhoea to pseudomembranous colitis, toxic megacolon, intestinal perforation and septic shock [6]. Nevertheless, the incidence and severity of *C. difficile* infections (CDIs) are not completely understood in this population [7]. Some reported risk factors for CDIs include advanced age, continued use of antibiotics, use of proton pump inhibitors and prolonged hospitalization [8, 9].

The literature has few reports on the occurrence of CDI in transplant paediatric patients, despite the fact that transplant recipients should be more susceptible to CDI because of multiple hospitalizations and immunosupression [10, 11]. A retrospective review of an institutional database in Germany reported CDIs in children after liver and kidney transplant without strain characterization [12]. Molecular characterization reveals how a strain evolves and is transmitted, and it is also important for surveillance and infection control of CDI. Here, we report a case of CDI in a child who received a liver transplant, including molecular characterization of the *C. difficile* strain.

## Case report

A 2.5-year-old girl with a history of liver transplantation at 7 months of age was admitted with diarrhoea at Albert Sabin Children’s Hospital in Fortaleza, Brazil. The stool was bloody and mucoid, with an average frequency of six bowel movements per day, which was unrelated to diet, but was associated with diffuse abdominal pain. There was no vomiting. Physical examination revealed that the patient was in good general condition and afebrile, eupneic, hydrated, anicteric, acyanotic and without cardiopulmonary alterations. Her abdomen was distended but soft and non-tender, with the liver edge 3 cm below the right costal border and the spleen 4 cm below the left costal border. Thorax percussion gave normal results on auscultation.

The compete blood count revealed leukocytosis with neutropenia and lymphocytosis (10 420 mm^−3^, neutrophils 24 % and lymphocytes 53 %), low haemoglobin (10.5 g dl^−1^), normal platelets (191 600 mm^−3^) and normal renal function (urea 26 ml dl^−1^ and creatinine 0.2 mg dl^−1^). We did not detect intestinal parasites (i.e. protozoa and helminths), gastrointestinal viruses, gastrointestinal viruses, or enteric pathogens in the patient’s stool sample.

The patient had three prior diarrhoeal illnesses. Her past medical history was notable for biliary atresia diagnosed when she was 3 months of age. A liver transplant was performed when she was 7 months old, and she has been taking immunosuppressive medication (prednisone/tacrolimus/mycophenolate sodium) since that time. No proton pump inhibitor was prescribed.

At our hospital, an initial screen for *C. difficile* toxins is performed for all patients with diarrhoea who have risk factors. A stool sample was examined on the third day of the onset of diarrhoea that was positive for *C. difficile* toxin A/B (we used ProSpecT *Clostridium difficile* Toxin A/B Microplate Remel). We cultured the sample on selective CCFA media (cefoxitin–cycloserine–fructose agar, Oxoid) as well as on fastidious anaerobe broth (FAB), over 5 and 15 days, respectively. We incubated the samples in anaerobic conditions (90 % N_2_, 10 % CO_2_). The FAB-positive broth was inoculated onto another CCFA media plate. Characteristic *C. difficile* colonies appeared on CCFA [13]. These were tinged with yellow colour, and presented a ground-glass appearance characterized by circular elements with slightly filamentous edges. These were flat to low with rounded elevations. We inoculated the colonies onto *Brucella* agar with 5 % lysed sheep blood and vitamin K (5 mg ml^−1^) to identify them further and to perform molecular tests.

The *C. difficile* isolates were confirmed using the RapID ANAII system (Remel) and by PCR amplification [14]of the *tpi* gene (* C. difficile* housekeeping gene).

Using a previously reported method [14], we obtained positive results for amplification assays for *tcdA* and *tcdB* gene fragments of PaLoc. By contrast, we obtained negative results for the binary toxin gene *cdtB* as well as the putative regulatory gene *tcdC*. We conducted molecular strain typing by amplification of the 16S–23S rRNA intergenic spacer sequences [15] and pulsed-field gel electrophoresis (PFGE) with *SmaI* genomic DNA digestion, as described previously [14]. PCR ribotypes were determined by subjecting data to the database WEBRIBO (http://webribo.ages.at). We compared DNA fragment patterns to those in the database of the National Microbiology Laboratory, Canada, using BioNumerics software (version 5.1. Applied Maths). We designated the isolate as PCR ribotype 046 and the new pulsotype as 1174 .

We determined the antibiotic susceptibility profile using the E-test (bioMérieux) on *Brucella* agar supplemented with vitamin K (5 mg ml^−1^) and 5 % defibrinated sheep blood. The isolate was resistant to levofloxacin (4 µg ml^−1^) and susceptible to vancomycin (1.5 µg ml^−1^), metronidazole (0.38 µg ml^−1^), rifampicin (<0.002 µg ml^−1^), moxifloxacin (1.0 µg ml^−1^) and clindamycin (1.0 µg ml^−1^). Resistance breakpoints were set in agreement with the guidelines of the Clinical Laboratory Standard Institute (CLSI) (M11-A8). For levofloxacin, we used the moxifloxacin breakpoint; for vancomycin, we used the EUCAST guidelines (http://www.eucast.org/clinical_breakpoints/); and for rifampicin, we used the breakpoint given by O'Conner *et al.* [16].

The diarrhoea ceased on the fourth or fifth day after the initiation of therapy with metronidazole (7.5 mg kg^−1^ three times a day for 14 days). No probiotic therapy was used.

## Discussion

Here, we report a case of a child with a history of hospitalization, antibiotic administration, hepatic transplant and immunosuppressive medication therapy, all of which are risk factors for CDI. A recent study reported that 92 % of children with CDI had a history of previous antibiotic use, 60 % were on immunosuppressive treatment, 39 % had malignancy or underwent organ transplantation and 13 % had inflammatory bowel disease [17].

A retrospective review of institutional databases showed that children undergoing solid organ transplant (SOT) accounted for 20 % of all detected CDIs in immunocompromised paediatric patients in Germany [12]. These data reinforce the importance of inquiry for CDI in children after SOT in developing countries [18, 19].

The isolated 046 ribotype strain was toxigenic (*tcd*A and *tcd*B gene fragments of PaLoc were positive), which explains the diarrhoea. PCR ribotype 046 has previously been isolated from neonatal pigs and humans in Sweden, indicating its zoonotic potential [20], and in adult tuberculosis patients in Poland, where a highly rifampicin-resistant PCR ribotype 046 clone was isolated [21]. PCR ribotype 046 has also been described as causing adult CDI in Chile [22]. Despite the fact that this ribotype has been isolated in a healthy child in southeast Brazil [23], ours is the first report of this ribotype causing paediatric diarrhoea.

The isolation and identification of strains has been a crucial practice in hospital services to provide a better understanding of the severity, recurrence and antibiotic resistance of CDIs. Although some studies have shown that ribotype 046 is resistant to antibiotics such as amoxicillin, rifaximin and erythromycin [21, 23], the strain isolated in this study did not show antibiotic resistance, except for levofloxacin. Our patient responded to treatment with metronidazole, which is the first-line agent for initial and first recurrence of mild-to-moderate severity in paediatric patients with CDIs [1]. Most children with CDIs are treated with a single agent, typically metronidazole (53–63 %) [24].

This case shows the importance of considering CDI in children following SOT in developing countries. The case highlights the relevance of strain molecular characterization to understand epidemiological spread as well as clinical features. Because CDI prolongs hospitalization time, increases healthcare costs, and can cause death, vigilance in health care units for CDI in the paediatric population, notably in immunosuppressed children after SOT, should be emphasized.
